# Discrimination and Social Exclusion in the Outbreak of COVID-19

**DOI:** 10.3390/ijerph17082933

**Published:** 2020-04-23

**Authors:** Jun He, Leshui He, Wen Zhou, Xuanhua Nie, Ming He

**Affiliations:** 1 School of Ethnology and Sociology, Yunnan University, Kunming 650092, China; jun.he@ynu.edu.cn (J.H.); zwen03@163.com (W.Z.); niexuanhua1988@ynu.edu.cn (X.N.); 2 Department of Economics, Bates College, Lewiston, ME 04240, USA; lhe@bates.edu

**Keywords:** discrimination, social exclusion, coronavirus

## Abstract

This paper is aimed to document the observed social exclusion and discrimination in the outbreak of COVID-19 across the world and inside of China. Discrimination and social exclusion has occurred in various forms, while 25.11% of respondents overseas experienced discrimination in the breakout of COVID-19, and 90% of respondents inside of China exhibited discriminatory attitudes. The discrimination and social exclusion also lead to a range of damaging social outcomes. Thus, this is an urgent call for the inclusiveness in policy and media in the face of this public health emergency.

## 1. Introduction

The COVID-19 virus, previously known as 2019-nCov, was first found in late December 2019 in Wuhan, Hubei Province, China [1], before it rapidly spread across the entire country and then the world. As of 15 April 2020, there are over 1.9 million confirmed cases, with at least 123,000 deaths across 213 countries [2]. Since scientists confirmed the possibility of person-to-person transmission [3,4], massive efforts have been undertaken to isolate confirmed and suspected cases from the public. In particular, as learned from previous experiences in controlling the spread of SARS, the lockdown approach was widely applied, and has now been proven for its effectiveness in controlling transmission [5]. While these efforts are designed to limit further infection, instances of discrimination (Here, we take the general definition of discrimination to refer to unjust treatment or making a distinction against a person based on group, class, race, age, sex or category to which that person belongs rather than on individual merit.) and social exclusion have also increased as the number of reported confirmed cases continues to rise. This research is aimed to document the observation and unexpected social outcomes of social exclusion and discrimination in the outbreak of COVID-19 across the world and inside of China. 

## 2. Methodology

The data generated from this research are delivered from three primary sources. (1) An online survey targeted at oversea Chinese people was conducted in February 2002 via the snowball sampling method. We used an online survey platform (‘SurveyStar,’ Changsha Ranxing Science and Technology, Shanghai, China) to disseminate questionnaires firstly to friends and students who would pass it on to others. The survey finally reached 1904 Chinese residents overseas across 70 countries. (2) Similar to the survey on Chinese people overseas, an online survey targeting Chinese people in mainland China collected 17,846 responses across 31 provinces. (3) Secondary data from newspaper, Internet and other sources of documentation were collected to gather descriptive data for this research. These data collection efforts were particularly designed to avoid person-to-person contact. 

## 3. Observation of Discrimination and Social Exclusion 

Globally, people of Chinese or Asian descent have overwhelmingly been the target of discrimination and social exclusion. Since the outbreak started in January, numerous news outlets around the world have reported cases of discrimination against those with Asian descent occurring in public—on the bus, in shopping malls, on the street and on school campuses. On January 31st in Sheffield and in Berlin, February 5th in New York, February 7th in Toronto and many other cases in Australia and Europe, incidents of discrimination took place against Chinese women wearing facemasks [6,7,8]. The range of discriminatory acts varied from verbal abuse to violent attacks. An important component of such discrimination is that, prior to the recent months, face mask-wearing had implied sickness in the West, while such behavior is ubiquitous to the daily life of many Eastern Asian countries to an extent that it has even been viewed as a fashion statement. This cultural difference and the legacy of racism may have interacted to contribute to the increased discrimination. 

Discriminatory behavior is often unobservable. We conducted a global survey in February 2020 that reached 1904 Chinese residents overseas across 70 countries. Of the respondents, 25.11% reported to have experienced different forms of discrimination, including being laid off without proper cause, rejection of rental housing and commonly reported abuses in the public (Supplementary Materials: Oversea). Strikingly, most respondents who experienced such discrimination are more likely to reside in high-income countries (see Table 1). Table 1 shows that women, youths and those who are less educated are more likely to experience discrimination and even violent overactions, while people with permanent resident status are less likely to report such experiences. Interestingly, as shown in Table 1, respondents living in the countries with more confirmed case of COVID-19 are less likely to report cases of discrimination and overaction. 

At the same time, increased social exclusion of those from areas most impacted by the virus also took place within racial and national boundaries. In China, many fear contact with people from Wuhan or Hubei Province. The stigmatization of people from Hubei is associated with the social exclusion process. For instance, in Yunnan Province, a popular tourist destination that hosted millions of tourists in January 2020, hotels turned away pre-booked guests from Wuhan or Hubei, regardless of their health condition. Across the country, both urban and rural communities have set up check-points to block visitors from Wuhan and Hubei without a medical check. Moreover, many local authorities have required their residents to report travel to Hubei or any physical contact with residents from Hubei Province. Cars with registration from Hubei have also been regarded as virus carriers and have been attacked in many provinces. In a different survey that we conducted that reached over 17,846 participants across 31 provinces in mainland China (including autonomous regions, special administrative regions and municipalities), almost 90% of respondents suggested that they would report to local authorities if any people from Hubei appeared in their community, 50.58% of respondents would avoid people from Hubei and 16.94% would even actively expel those people from Hubei from their communities (Supplementary Materials: Domestic). 

Discrimination and social exclusion may lead to damaging social outcomes, especially in the face of infectious diseases. While increasing the resistance to the demonization of those of Chinese or Asian descent in Western countries may lead to more abuse and violence at the global level, discrimination and social exclusion can undermine efforts to identify, isolate and contain the transmission of the virus. Potential carriers traveling from infected areas, having been denied regular access to housing and food, may have to spend more time in search of these essential supplies, potentially turning to illegitimate sources, thus increasing contact with others and rendering them unable to effectively self-quarantine. Furthermore, social stigma reduces the likelihood of them coming forward for help, preventing medical practitioners from effectively containing and treating the disease at early stages. In extreme cases, patients might even attempt to escape from hospitals, as previously occurred during the outbreak of other infectious diseases like SARS, Ebola and HIV [9,10].

## 4. Concluding Remark

This fear of unknown diseases is a part of human nature, especially when they are deadly and highly infectious. Stigmatization of COVID-19 led by some politicians such as Donald Trump might have reinforced such discrimination and social exclusion, as what Foucault called “biopolitics.” However, it is paramount to recognize the discriminatory behaviors that accompany fear, as they damage not only the socio-cultural fabric in the long-run, but they also compromise present efforts to contain the disease. The Chinese government has begun acting to reduce discriminatory practices by fostering understanding and support in the media, alongside its recommendations to reduce travel and interpersonal contact. In addition, the government has increased support for Hubei residents by providing government-funded hotel rooms and free healthcare for COVID-19 virus treatments in all provinces across China. Internationally, universities in the UK like Sussex University have started to provide support for Chinese students to limit abuse and discrimination, while the UK government is also attempting to track any form of COVID-19 associated crimes. While those efforts to eliminate discrimination and social exclusion are a good start, further measures are still required, particularly post-pandemic. Thus, in the face of this public health emergency, there is an urgent call for the inclusiveness of policy and media. 

## Figures and Tables

**Table 1:** Logit model of discrimination and overaction across the world.

	Discrimination	Violent Overaction
Age	−0.153 ** (0.055)	−0.190 *** (0.051)
Gender (female = 0)	0.039 (0.115)	-0.121 (0.107)
Education	−0.181 ** (0.069)	−0.090 (0.066)
Residential statute (Non-permanent = 0)	−0.518 *** (0.131)	−0.128 (0.122)
Log infected Number	−0.134 ** (0.052)	−0.326 *** (0.050)
Income level of the country	0.448 *** (0.105)	0.542 *** (0.099)
Constant	−0.876 (0.450)	−0.832 * (0.415)
Observations	1904	1904
Pseudo- R2	0.028	0.031
Log likelihood	−1036.045	−1132.301

Standard errors in parentheses; * *p* < 0.05, ** *p* < 0.01, *** *p* < 0.001. Notes: Coefficients (log odds) from ordered logit models are presented. Numbers in parentheses are standard errors. Income level of country is based on World Bank classification in current 2020 fiscal year. Infected number is based on World Health Organization data on 17 February 2020.

## Supplementary Materials

The following are available online at https://www.mdpi.com/1660-4601/17/8/2933/s1, Oversea: Overseas Chinese experienced discrimination and violent overaction; Domestic: reaction when encounter Hubei people by mainland Chinese.
